# Effects of Lipid Regulation Using Raw and Processed Radix Polygoni Multiflori in Rats Fed a High-Fat Diet

**DOI:** 10.1155/2012/329171

**Published:** 2012-12-09

**Authors:** Na Li, Zhen Chen, Xiaojian Mao, Jie Yu, Ronghua Zhao

**Affiliations:** ^1^Yunnan University of Traditional Chinese Medicine, Kunming, Yunnan 650500, China; ^2^Medicine Division, Yunnan Baiyao Group Co. Ltd., China

## Abstract

Raw and processed Radix Polygoni Multiflori have been used in the prevention and treatment of nonalcoholic fatty liver disease (NAFLD), hyperlipidemia, and related diseases in Asian counties for centuries. The lipid regulation ability of raw and processed Poligoni Multiflori Radix were compared in high-fat diet fed rats in this research. Total cholesterol (TC) and low density lipoprotein-cholesterol (LDL-C) in blood and liver tissue were all significantly higher in model rats. However, triglyceride (TG) contents increased only in liver tissue, not in the blood samples. The rats fed the high-fat diets were considered the model of type IIa hyperlipidemia and early-stage nonalcoholic fatty liver disease. Both Radix Polygoni Multiflori (RPM) and Radix Polygoni Multiflori Praeparata (RPMP) revealed TC-lowing effects, and middling doses of RPMP displayed the most significant TC-lowing effects, as indicated by blood samples. Neither RPM nor RPMP was found to reduce LDL-C in rats' blood. Nevertheless, RPM showed dose-dependent TC- and TG-lowing effects in the liver tissue samples. In conclusion, RPM showed more pronounced effects on lipid regulation in liver samples in the treatment of early-stage NAFLD. RPMP, however, displayed better effects in regulating lipids in circulating blood for the treatment of hyperlipidemia.

## 1. Introduction

Fatty liver is a reversible condition in which large vacuoles of triglyceride fat accumulate in liver cells via the process of steatosis (abnormal retention of lipids within a cell). Considering the contribution that alcohol can make to this condition, fatty liver may be termed alcoholic steatosis or non-alcoholic fatty liver disease (NAFLD).

The development of nonalcoholic fatty liver disease comes from an imbalance between the influx and production of fatty acids and the use of fatty acids for oxidation or secretion. The progress of NAFLD is usually characterized by morphologic changes in the hepatocytes and hepatic triglyceride content (HTGC). However, early-stage NAFLD shows changes only in the fatty acid levels but no or few morphologic changes in hepatocytes [1]. In normal human livers, the mean values for total cholesterol (TC) and triglyceride (TG) are 3.9 and 19.5 mg/g wet weight, respectively. Hepatic steatosis (fatty liver) is a condition that is defined by fat accumulation within hepatocytes that exceeds 50 mg/g of the liver by weight [2, 3].

Fatty liver disease was recently recognized as a feature of the metabolic syndrome; fatty liver has evolved as a key player in the pathogenesis of hyperlipidemia. Hyperlipidemia is characterized by elevated TG, TC, and low-density lipoprotein cholesterol (LDL-C) and decreased high-density lipoprotein cholesterol (HDL-C) levels. Hyperlipidemia is a major risk factor for cardiovascular disease.

Because of the effectiveness and acceptable prices, the prevention and treatment of NAFLD and hyperlipidemia by traditional Chinese medicine attract more and more attention worldwide [4, 5]. Radix Polygoni Multiflori (RPM, heshouwu in Chinese) and Radix Polygoni Multiflori Praeparata (RPMP, zhiheshouwu in Chinese), originating from the root of *Polygonum multiflorum* Thunb., are used in the treatment of NAFLD and hyperlipidemia in oriental counties for centuries [6]. Although both RPM and RPMP have a history of use in the treatment of NAFLD and hyperlipidemia, RPMP is used more frequently in traditional Chinese medicine than RPM is. For example, the Pharmacopoeia of the People's Republic of China (2010 edition) lists three different prescription preparations containing RPMP for the treatment of hyperlipidemia [6]: Xuezhining Wan, Xuezhiling Pian, and Shouwu Wan. It lists only one preparation, Zhengxin Jiangzhi Pian, that contains RPM. In the meantime, few studies have compared the effects of RPM and RPMP in the treatment of NAFLD, hyperlipidemia, and related diseases.

Our research group has performed previous studies including systematic comparisons of raw and processed RPM with respect to their antioxidative activities, adverse laxative effects, cytotoxicity, and *in vitro* lipid-regulation effects [7–9]. In our *in vitro* studies, raw RPM showed stronger abilities to regulate TG and TC than RPMP, indicating that RPM might be effective in the clinic treatment of NAFLD. However, *in vivo* results are required for corroboration. We compared the relative activities of raw and processed RPM in SD rats fed high-fat diets. Comprehensive studies of indexes of lipid metabolism in both the blood and liver tissue samples of the test animals were analyzed.

## 2. Materials and Methods

### 2.1. Chemicals

Simvastatin (Hangzhou MSD Pharmaceutical Co., Ltd., China) and fenofibrate (Laboratories Fournier S.A., France) were used as positive controls for lowering cholesterol and triglyceride levels, respectively. Lard oil was purchased from the Shuangliu Luxiao oil factory in Chengdu, Sichuan Province, China. Sodium chloride, methylthiouracilum (MTU), ether, and other reagents were of analytical grade.

### 2.2. Processing and Extraction of Radix Polygoni Multiflori

**Figure 1:**
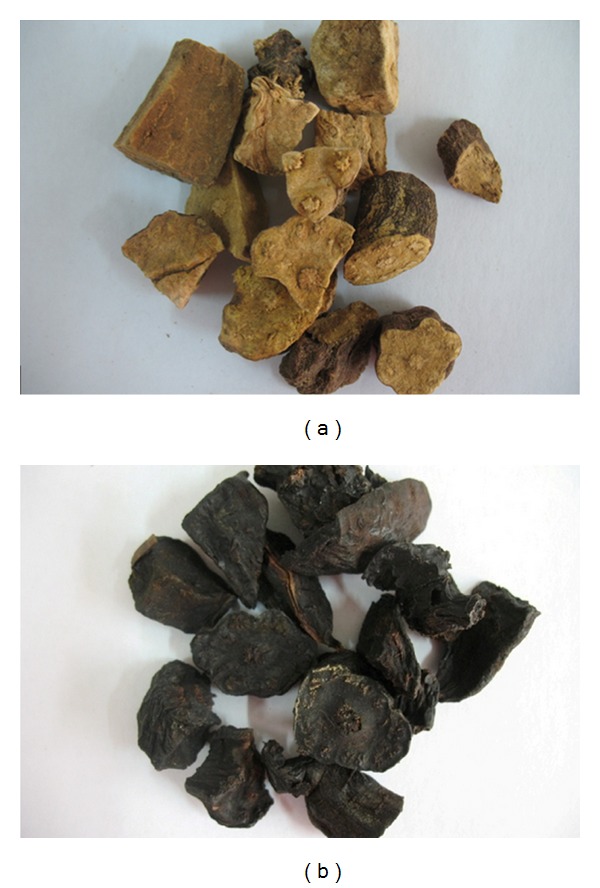
Photographs of raw (a) and processed (b) Radix Polygoni Multiflori.

*Polygonum multiflorum* Thunb. plants were collected in June of 2008 by the authors in Luquan County within Yunnan Province and identified by Professor Ronghua Zhao of Yunnan University of Traditional Chinese Medicine. No specific permits were required for the described field studies. This location is not privately owned or protected in any way and the field studies did not involve endangered or protected species. Voucher specimens were deposited in the Herbarium of Pharmacognosy, Yunnan University of Traditional Chinese Medicine. RPMP was steamed by the authors from RPM with black soybean decoction according to the procedure recorded in Pharmacopoeia of the People's Republic of China (2010 edition) (Figure 1) [6].

The main components of RPM and RPMP were TSG (2,3,5,4′-tetrahydroxy-stilbene-2-O-*β*-D-glucoside), emodin, and physcion. The content of TSG was lower in RPMP than RPM. However, the contents of emodin and physcion were increased after processing [8, 9].

Extracts, 300 g RPM and 472 g RPMP, were decocted with water (10 times, 8 times, and then 6 times by volume) for three times, respectively. Extracts were combined, condensed, and lyophilized. The concentrations of RPM and RPMP were 0.6980 g/mL and 0.8580 g/mL, respectively. The recommended dosages of RPM and RPMP are 3–6 g and 6–12 g/per day according to the Pharmacopoeia of the People's Republic of China, 2010 edition [6]. We conversed the human dosage equivalently to rat dosage. The low, middle, and high dosages of RPM are 0.405, 0.810, and 1.62 g/kg body weight [10]. The low, middle, and high doses of RPMP are 0.810, 1.62, and 3.24 g/kg body weight.

### 2.3. Animals and Diets

SD rats of both sexes were provided by Experimental Animal Center of Yunnan University of Traditional Chinese Medicine. They were aged 8 weeks and weighed 245 ± 20 g. Rats of the same sex were housed six to a stainless steel cage containing sterile paddy husk as bedding in ventilated animal rooms. They were acclimated in the controlled environment (temperature 22 ± 1°C; 60 ± 10% humidity; and a 12 h/12 h light/dark cycle) with free access to water and a commercial laboratory complete food. All animal experiments were performed in compliance with the animal experimental ethics committee of Yunnan University of Traditional Chinese Medicine. All reasonable efforts were made to minimize the animals' suffering.

Diets designed to meet the nutritional requirements of rats were purchased from Suzhou Shuangshi Laboratory Animal Feed Science Co., Ltd., China. The high-fat diets contained 1% cholesterol, 10% lard, 0.2% methyl thiouracil, and 88.8% usual feed (moisture: ≤10%; protein: ≥20%; fat mix: ≥4%; calcium: 1.0–1.8%; phosphorus: 0.6–1.2; fiber: ≤5%; essential amino acids: ≥2%) and were prepared by the authors. The diet recipe was a classic formulae for the establishment of hyperlipidemia, non-alcoholic fatty liver disease (NAFLD), and related diseases recorded in Pharmacological Method (Third edition) [11].

One hundred and twenty SD rats of both sexes were randomly divided into 10 groups of twelve each (Table 1). The male and female rats were all six for each group. Group A received normal diets only and served as vehicle. Group B received high fat diets only and served as the model group. Other groups were fed high fat diets throughout the whole study periods. Eighteen days after the start of the study, the RPM group (Groups C, D, and E), RPMP group (Groups F, G, and H), and two positive control groups (simvastatin in Group I and fenofibrate in Group J) received corresponding treatments every day for another 24 days (Table 1). All rats were fasted for 2 h every day before administration of therapeutic agents.

### 2.4. Assessment of Total Cholesterol, Triglyceride, Lipoprotein, and Liver Marker Enzyme in Blood

Samples of blood 1.5–2 mL in volume were collected from the retro-orbital venous plexus once every 6 days throughout the study. Blood Samples were collected under ether anesthetic condition, two hours after administration of therapeutic agents in the morning. Serum was centrifuged at 16,000 rpm for 15 min and analyzed immediately. Levels of AST, ALT, TG, TC, LDL-C, and HDL-C in serum were determined by enzymatic colorimetric method using commercial standard enzymatic assay kits (Biosino Bio-technology & Sience Inc.) by AB-1020 automatic biochemical analyzer (Sunostik Medical Technology Co., Ltd.). TG and TC were tested on days 0, 6, 18, 24, 30, 36, and 42. LDL-C and HDL-C were tested on days 0, 6, 18, 24, 30, and 42. AST and ALT were tested only on days 0, 18, and 42 day. VLDL was analyzed using enzyme-linked immunosorbent assay kits from Rapidbio (U.S.). VLDL was tested only on days 0, 18, and 42. All bioassays were carried out in duplicate.

### 2.5. Assessment of Total Cholesterol, Triglyceride, Lipoprotein, and Liver Marker Enzyme in Liver Tissue

The rats were sacrificed by cervical dislocation two hours after the last administration of therapeutic agents in the morning. Tissue samples from their livers were immediately processed for biochemical analysis and morphologic observations. Single-gram tissue samples of liver were homogenized with 9 mL ice-cold 0.9% physiological saline. Homogenates of liver were centrifuged at 4000 rpm for 10 min at 4°C. Five milliliters of supernatant was maintained at −80°C until analysis. One hundred microliter samples of liver homogenate were diluted with 400 *μ*L distilled water for determination [12]. AST, ALT, TG, TC, LDL-C, and HDL-C were tested in all liver tissue samples at the end of the study.

### 2.6. Morphologic Observations

For light microscopic observations, samples from liver were fixed in formalin fixative and processed routinely for embedding in paraffin. Tissue sections 5 *μ*m in thickness were stained with hematoxylin and eosin (H&E) and examined under a light microscope.

### 2.7. Statistical Analysis

All data in this study are expressed in the form of mean ± SD. The data were evaluated by one-way analysis of variance (ANOVA), and the differences between means assessed using Duncan's test with a significance level of *P* < 0.05, <0.01, and <0.001.

## 3. Results

### 3.1. General Condition of Rats

**Figure 2:**
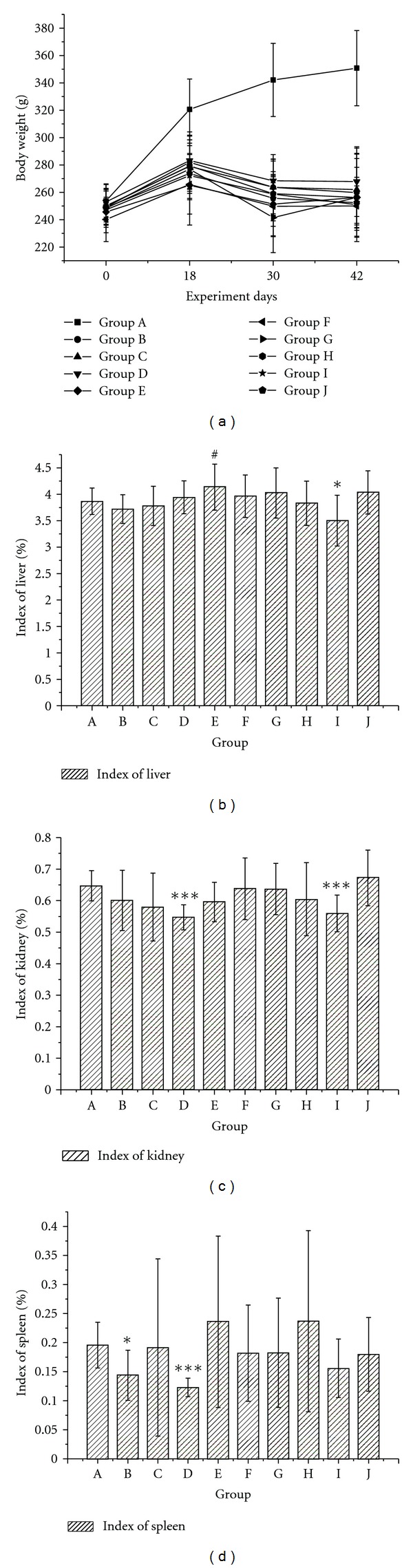
Body weight (a) and indexes of liver (b), kidney (c), and spleen (d) of rats in different groups. Note: The body weight was measured in every five days, however, only the data in 0th, 18th, 30th, and 42nd day were listed in this figure. The * indicates a significant difference compared with control group, *P < 0.05 and ***P < 0.001. The ^#^ indicates a significant difference compared with model group, ^#^ *P* < 0.05.

One hundred and twenty Sprague-Dawley rats of either sex were randomly divided into 10 groups of twelve in each (Table 1). The rats fed on normal diets showed about 43% weight gain in 42 days of experiment duration (Figure 2(a)). However, the body weights of rats fed high-fat diets were significantly lighter than those of rats fed normal diets groups beginning on day 18 day and persisted until the end of the experiment across all treatment groups (Figure 2(a)). We attributed the weight loss to the rats' displeasure with the taste of the high-fat diets.

Indexes of liver, kidney, and spleen were recorded after the rats were killed (Figures 2(b), 2(c), and 2(d)). Liver and spleen indexes showed no significant differences between the control group and the high-fat-diet group. However, kidney indexes in the high-fat diet group were slightly lower than those of the in control group. Slight decreases in the kidney and spleen indexes were observed in the middle-dose RPM (Group D). The liver and spleen indexes of rats in the simvastatin group also decreased. Indexes of liver lipid levels were increased in the high-dosage RPM group (Group E). No serious pathological alterations were observed in this study, as determined by organ indexes.

### 3.2. Biochemical Indexes in Blood Sample of Model Group Rats

The TG contents in Group B were higher than those of Group A on day 6. After that, the TG contents dropped to a level lower than Group A (Table 2). The TC contents in Group B increased acutely from the beginning of the experiment (Table 3). The TC contents of Group B were increased to the highest level on day 24, 359.9 ± 61.90 mg/dL, almost 3 times than control group (Table 3). The amplitude of TC contents started to be decrease after day 24 but remained very high, at about 300 mg/dL.

LDL-C (Table 4) and HDL-C (Table 5) contents in Group B were higher than Group A from day 6 through the end of the study. Very low density lipoprotein (VLDL) content, as indicated by ELISA kits, showed no differences from the control group at the beginning of the study or on day 18 (Table 6). At the end of the experiment, the VLDL contents were even lower than those of control group.

Blood levels of aspartate aminotransaminase (AST) and alanine aminotransaminase (ALT) were evaluated on days 0, 18, and 42 (data not shown). No increases in AST or ALT were observed, which indicated that liver damage did not occur during the research period.

### 3.3. Biochemical Indexes in Liver Tissues of Model-Group Rats

**Figure 3:**
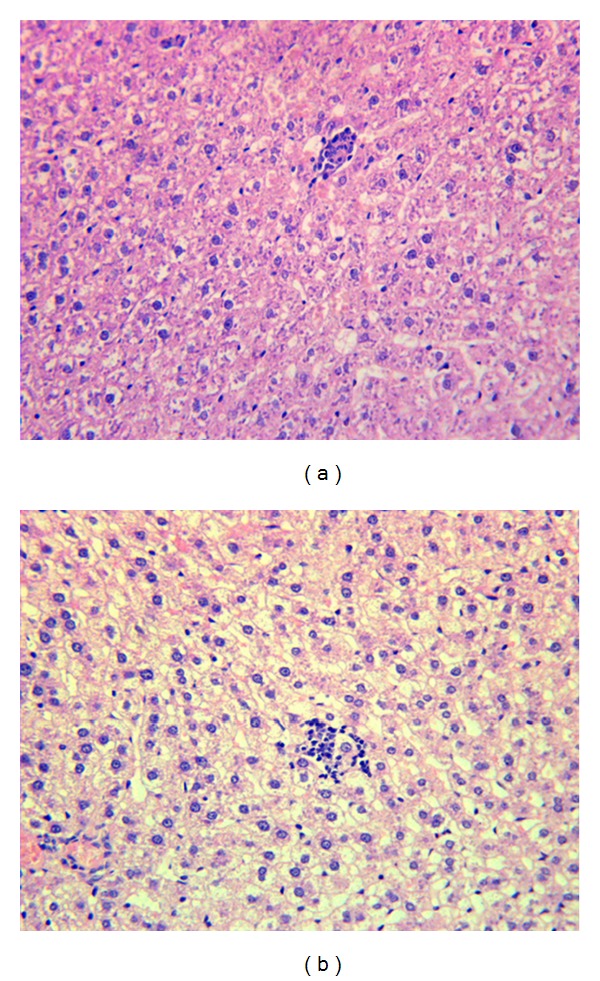
Comparison of microscopic morphology in liver tissue between control group (a) and model group (b). Intracellular edema was widely distributed in liver tissue in model group; however, fatty drops were not observed in model group possibly due to the limited research time. Original magnification: 400x.

Levels of TG, TC, LDL-C, HDL-C, AST, and ALT contents in liver tissue were tested after execution at the end of the research (Table 7). TC, TG, and LDL-C levels in the high-fat diet group were significantly higher than in the control group, but other indexes showed no difference. The control and high-fat diet groups showed similar AST and ALT levels, indicating normal liver function. Morphologic observations were carried in every group. Livers in the high-fat diet groups were more paler than normal groups (figure not shown), probably due to the intracellular edema, However, no fatty deposits were observed in the liver biopsy slides of the model group (Figure 3). Actually, no fatty deposits were observed in normal diet group, high fat diet group, positive groups, RPM group, and RPMP groups (figure not shown). That is probably due to the detention of morphologic changes compared to the biochemistry changes. For these reasons, the model group rats were considered to have early-stage NAFLD.

According to the Fredrickson's classification of hyperlipidemia, type IIa hyperlipidemia is characterized by elevation of total cholesterol and LDL-C, while type IIb hyperlipidemia is characterized by elevation of total cholesterol, LDL-C, and VLDL-C. Judging from the lipid indexes listed above, the rats fed the high-fat diets were diagnosed with type IIa hyperlipidemia and early-stage non-alcoholic fatty liver disease.

### 3.4. Antihyperlipidemic Effects of RPM and RRPM

The TG levels in the blood samples were significantly higher in Group B after 6 days on the high-fat diet than those of control rats. TG did not continue to increase. TG levels were lower than those of control rats during the rest of the study. Considering the unremarkable increases in TG in the model group, we did not conclude that there was a relationship between different treatments, dosages, and durations of high-fat diet.

The RPM, RPMP, and positive control treatments began on day 19 of the study. No TC melioration effects were observed in the low-dosage raw RPM group. Both the middle (Group D) and high dosage (Group E) RPM groups showed significant changes in TC regulation in blood samples. The most pronounced TC regulation effects of RPMP were observed in the middle-dosage RPMP group (Group G). Time-dependent TC regulation activities were observed in Group G. At the end of the experiment, the TC levels in Group G were reduced to 188.0 ± 50.93 mg/dL, significantly lower than that of model group (*P* < 0.001).

Similar TC-lowing effects were observed between Group I (simvastatin group), Group D, and Group G during the early stages of treatment (from day 19 through day 30). However, the rats given traditional Chinese medicines showed better results than those given simvastatin at the end of the study (Group D, E, F, and G).

Dosage relationships were observed between the LDL-C lowing activity in RPM groups, although no significant differences were observed across different groups (Table 4). Simvastatin (Group I) and fenofibrate (Group J) were found to remarkably downregulate the LDL-C content by the end of the study (*P* < 0.05).

HDL-C contents were generally higher in rats fed a high-fat diet than those fed a normal diet no matter what kinds of treatment were administered (Table 5).

### 3.5. Anti-Nonalcoholic Fatty Liver Disease Effects of RPM and PMPR

Liver tissue samples were homogenized and analyzed in order to evaluate the lipid regulation activities of raw and processed RPM in the liver. The RPM showed significant abilities to reduce levels of hepatic TG and TC. High doses of RPM were found to thoroughly control the progress of fatty accumulation in the liver. The TG and TC contents dropped to 153.6 ± 27.34 mg/dL and 57.18 ± 6.754 mg/dL, respectively, which were similar to the levels observed in the control group. Dose activity relationships were observed in the RPM groups. However, PRMP dosage was not found to be directly related to lipid regulation effects. Low and high doses of RPMP showed better effects than middle doses.

No remarkable increases in AST or ALT were observed in any of the treatment groups, indicating that liver function remained normal.

Simvastatin (Group I) and fenofibrate (Group J) showed excellent abilities to reduce TC levels, but simvastatin even showed more pronounced abilities to reduce TG than fenofibrate.

LDL-C contents were increased in RPM and RPMP treatment groups, especially the RPMP treated group. Simvastatin (Group I) and fenofibrate (Group J) were both found to downregulate LDL-C content.

## 4. Discussion

Hyperlipidemia and NAFLD are major risk factors for cardiovascular disease. Certain frequently used lipid-lowing drugs (fibrates, stains, and bile acid sequestraints) used for the treatment of hyperlipidemia and NAFLD have numerous side effects. For this, traditional herb drugs with remarkable effect and lower price seem to be the best option. The prevention and treatment of hyperlipidemia and NAFLD through traditional Chinese medicine have attracted more and more attention worldwide.

In our previous studies, RPM showed more pronounced effects on TG and TC regulation in *in vitro* assays. This contradicted the widespread use of RPMP in treatment of hyperlipidemia and NAFLD. In this study, lipid regulation activities of RPM and RPMP were compared in both blood and liver tissue samples from rats fed a high-fat diet. *In vivo* hyperlipidemia and early-stage non-alcoholic fatty liver disease were induced in rats by feeding them high-fat diets for 18 days. These high-fat diets were continued through the 24 days of the experiments. Increases in the TC, LDL-C, and HDL-C levels of the blood samples and the TC, TG, and LDL-C levels of the liver tissue indicated the formation of hyperlipidemia and early-stage non-alcoholic fatty liver disease.

The low, middle, and high doses of RPM and RPMP administered to test rats in this study were calculated using the corresponding dosages recommended for human clinical use in the Chinese Pharmacopeia, 2010 edition [6]. The recommended dosage of RPMP is two times higher than that of RPM in the *Pharmacopoeia of People's Republic of China* (2010 edition) [6]. Thus, the dosage of PRMR was two times high than that of RPM in the same dosage group in this research.

TC levels in the circulatory system increased after the rats were fed high-fat diets, and these increases could be inhibited by both RPM and RPMP treatments. Both low (0.8100 g/kg body weight) and middle (1.620 g/kg body weight) dosages of RPMP revealed better TC lowing activity than middle dosages of RPM (0.8100 g/kg body weight). Judging from these results, we recommend RPMP in the treatment of hyperlipidemia characterized by increased levels of TC.

Because TG levels did not show any regular pattern of increase or decrease in rats fed high-fat diets, the RPM and RPMP did not appear to have any TG-reducing effects. The increases in LDL-C levels in the blood of rats fed high-fat diets were not reversed by RPM or RPMP. This was probably because of the limited duration of the treatment. However, simvastatin and fenofibrate were both found to reduce LDL-C levels during the study.

Lipid accumulation in the liver is the major hallmark of NAFLD. The increasing trends of TC and TG in the liver tissue of the model group were reversed after administration of RPM and RPMP. Obviously, dose-dependent TC- and TG-reducing effects were observed in PMP groups. High doses of RPM were found to restore liver TC and TG levels to the normal values. However, no similar dose-dependent relationship was observed in RPMP-treated groups. Increases in LDL-C content were controlled by both simvastatin and fenofibrate. Although simvastatin showed significant TC- and TG-reducing effects, significant elevation of AST and ALT were observed.

## 5. Conclusion

The results of the present study demonstrated the lipid regulation properties of RPM and RPMP. Each of these compounds showed the most pronounced lipid regulation effects in different organs. RPM showed better effects in liver cells in the treatment of NAFLD. This is consistent with the results of our previous *in vitro* study, which was carried on steatotic human liver L-02 cells and showed that RPM extracts could regulate the lipid content within liver cell better than RPMP [9]. RPMP displayed better effects than RPM in lipid regulation in the circulatory system, indicating that RPMP would have more pronounced curative effects in the treatment of hyperlipidemia.

## Figures and Tables

**Table 1:** Animal grouping and treatments in this research.

Groups	Diets	Treatment (from the 19th day of the experiment)	Dosage (g/kg body weight)
A	Normal diets	Physiological saline	1 mL per rat
B	High fat diets	Physiological saline	1 mL per rat
C	High fat diets	Water extraction of RPM	0.4050
D	High fat diets	Water extraction of RPM	0.8100
E	High fat diets	Water extraction of RPM	1.620
F	High fat diets	Water extraction of RPMP	0.8100
G	High fat diets	Water extraction of RPMP	1.620
H	High fat diets	Water extraction of RPMP	3.240
I	High fat diets	Simvastatin	0.001200
J	High fat diets	Fenofibrate	0.03300

**Table 2:** Triglyceride (TG) content in blood samples.

Group	0D	6D	12D	18D	24D	30D	36D	42D
A	68.64 ± 17.17	122.1 ± 25.29	105.2 ± 37.06	97.09 ± 23.32	50.54 ± 13.77	60.36 ± 18.54	136.2 ± 44.40	94.54 ± 27.69
B	102.0 ± 25.19	196.8 ± 46.82***	28.80 ± 13.21***	51.08 ± 14.03***	35.40 ± 8.746**	34.89 ± 11.52**	80.00 ± 9.212**	66.00 ± 28.79*
C	76.63 ± 22.78^#^	157.6 ± 28.75^∗∗, #^	23.09 ± 2.023***	44.27 ± 10.29***	37.27 ± 12.58*	50.82 ± 18.25^#^	101.4 ± 37.04	79.64 ± 20.25
D	76.45 ± 18.23^#^	175.4 ± 46.39**	23.00 ± 1.907***	36.91 ± 9.268^∗∗∗, #^	34.67 ± 6.856**	47.88 ± 18.66	77.00 ± 14.70**	71.88 ± 19.09
E	94.33 ± 34.39*	148.4 ± 47.49^#^	22.42 ± 1.886***	31.60 ± 17.88^∗∗∗, ##^	37.20 ± 11.71*	41.00 ± 19.05*	84.22 ± 19.76**	72.56 ± 14.43*
F	57.36 ± 18.91^###,†^	104.1 ± 35.60^###,††^	55.00 ± 15.27^∗∗∗, ###,†††^	31.89 ± 8.978^∗∗∗, ##,†^	39.91 ± 14.88	41.78 ± 16.27*	72.44 ± 19.75^∗∗∗,†^	78.11 ± 12.92
G	56.00 ± 15.42^###,††^	144.1 ± 70.24^#^	46.45 ± 9.903^∗∗∗, ##,†††^	28.00 ± 13.03^∗∗∗, ###^	22.45 ± 7.634^∗∗∗, ##,††^	47.10 ± 11.31^#^	78.00 ± 31.65**	71.14 ± 15.34
H	54.73 ± 15.94^###,††^	203.7 ± 63.82^∗∗∗,†^	64.73 ± 15.97^∗∗, ###,†††^	29.45 ± 9.136^∗∗∗, ###^	26.18 ± 5.134^∗∗∗, ##,†^	52.09 ± 22.06^#^	101.1 ± 38.43	88.82 ± 20.89
I	47.08 ± 9.615^∗∗, ###^	139.4 ± 39.37^##^	44.00 ± 12.40^∗∗∗, #^	21.91 ± 3.360^∗∗∗, ###^	27.09 ± 6.188^∗∗∗, #^	34.18 ± 11.12***	93.91 ± 24.76*	86.18 ± 32.54
J	93.42 ± 37.48	119.3 ± 52.07^###^	34.08 ± 18.81***	31.64 ± 11.66^∗∗∗, ###^	41.45 ± 12.39	32.54 ± 4.034***	83.82 ± 26.78**	85.22 ± 22.16

Triglyceride (TG) contents were assayed by assay kits as described in the text.

Values are mean ± SD (*n* = 12) and expressed in mg/dL.

The * indicates a significant difference compared with control group, *P < 0.05, **P < 0.01, ***P < 0.001.

The ^#^ indicates a significant difference compared with model group, ^#^ *P* < 0.05, ^##^ *P* < 0.01, ^###^ *P* < 0.001.

The ^†^ indicates a significant difference compared with PMR group (same dosage level, F compared with C, G compared to D, H compared to E), ^†^ *P* < 0.05, ^††^ *P* < 0.01, ^†††^ *P* < 0.001.

**Table 3:** Total cholesterol (TC) contents in blood samples.

Group	0D	6D	12D	18D	24D	30D	36D	42D
A	79.06 ± 11.88	84.70 ± 20.02	66.45 ± 9.115	91.77 ± 11.27	122.9 ± 31.84	73.16 ± 12.32	61.99 ± 10.06	61.48 ± 10.08
B	84.12 ± 8.411	176.4 ± 36.61***	165.4 ± 27.44**	262.6 ± 45.02***	359.9 ± 61.90***	296.0 ± 80.12***	290.8 ± 66.87***	290.1 ± 46.93***
C	83.40 ± 18.30	159.2 ± 26.99***	184.6 ± 84.74***	261.7 ± 41.11***	360.2 ± 84.24***	294.5 ± 111.8***	328.8 ± 126.5***	239.7 ± 69.45***
D	85.43 ± 18.49	161.2 ± 56.09***	194.6 ± 26.80^∗∗∗, #^	257.0 ± 32.58***	247.3 ± 54.32^∗∗∗, ###^	244.8 ± 41.50***	192.8 ± 33.40^∗∗∗, ##^	227.3 ± 39.93^∗∗∗, #^
E	80.97 ± 8.531	178.7 ± 42.40***	187.5 ± 55.53***	255.1 ± 27.57***	247.8 ± 74.33^∗∗∗, ##^	221.8 ± 54.88^∗∗∗, #^	198.2 ± 45.39^∗∗∗, ##^	227.6 ± 28.22^∗∗∗, ##^
F	89.7 ± 10.92*	143.4 ± 30.45^∗∗∗, #^	168.0 ± 28.74***	291.8 ± 72.83***	232.1 ± 95.54^∗∗, ##,††^	209.0 ± 57.89^∗∗∗, #^	182.8 ± 47.29^∗∗∗, ##,††^	212.3 ± 43.93^∗∗∗, ##^
G	88.61 ± 20.01	140.3 ± 26.14^∗∗∗, #^	209.8 ± 54.84^∗∗∗, #^	293.4 ± 47.51^∗∗∗,†^	227.2 ± 57.10^∗∗∗, ###^	216.3 ± 47.35^∗∗∗, #^	186.9 ± 104.9^∗∗∗, #^	188.0 ± 50.93^∗∗∗, ###^
H	90.65 ± 26.83	244.4 ± 87.23^∗∗∗, #,†^	211.1 ± 33.37^∗∗∗, ##^	296.5 ± 73.34***	237.5 ± 44.76^∗∗∗, ###^	219.9 ± 58.06^∗∗∗, #^	262.6 ± 60.32^∗∗∗,†^	299.4 ± 70.86^∗∗∗,†^
I	82.85 ± 7.949	183.6 ± 55.04***	227.7 ± 42.07^∗∗∗, ###^	274.7 ± 60.25***	235.5 ± 63.81^∗∗∗, ###^	238.4 ± 82.48***	239.5 ± 43.00***	263.3 ± 53.51***
J	78.25 ± 10.91	134.6 ± 17.74^∗∗∗, ##^	160.9 ± 22.66***	237.9 ± 32.93***	243.7 ± 40.02^∗∗∗, ###^	259.5 ± 61.93***	237.4 ± 91.13***	250.9 ± 34.42***

Total cholesterol (TC) contents were assayed by assay kits as described in the text.

Values were mean ± SD (*n* = 12) and expressed in mg/dL.

The * indicates a significant difference compared with control group, *P < 0.05, **P < 0.01, ***P < 0.001.

The ^#^ indicates a significant difference compared with model group, ^#^ *P* < 0.05, ^##^ *P* < 0.01, ^###^ *P* < 0.001.

The ^†^ indicates a significant difference compared with PMR group (same dosage level, F compared with C, G compared to D, H compared to E), ^†^ *P* < 0.05, ^††^ *P* < 0.01.

**Table 4:** Low density lipoprotein cholesterol (LDL-C) contents in blood samples.

LDL	0D	6D	18D	24D	30D	42D
A	22.33 ± 7.896	15.63 ± 4.228	28.45 ± 8.157	23.03 ± 3.350	16.94 ± 3.410	16.28 ± 3.567
B	26.23 ± 7.143	68.03 ± 24.60***	67.22 ± 13.45***	83.22 ± 21.49***	88.98 ± 32.26***	98.80 ± 35.21***
C	23.24 ± 6.831	51.57 ± 14.85***	70.48 ± 17.22***	89.36 ± 44.32***	114.1 ± 62.90***	92.35 ± 52.31***
D	18.56 ± 5.616	57.10 ± 17.34***	49.71 ± 14.07^∗∗∗, ##^	69.78 ± 20.54***	88.17 ± 19.97***	80.05 ± 29.15***
E	21.98 ± 3.974	47.85 ± 17.38^∗∗∗, #^	51.16 ± 9.723^∗∗∗, ##^	60.05 ± 21.35^∗∗∗, #^	98.18 ± 38.69***	74.29 ± 29.43***
F	19.76 ± 2.608	42.43 ± 16.61^∗∗∗, ##^	50.66 ± 12.67^∗∗∗, ##,†^	76.53 ± 43.37***	87.61 ± 48.40***	73.00 ± 32.86***
G	26.94 ± 6.340^††^	36.67 ± 13.89^∗∗∗, ###,††^	47.81 ± 14.72^∗∗∗, ##^	109.1 ± 44.91^∗∗∗,†^	97.41 ± 40.59***	70.91 ± 63.17*
H	23.69 ± 3.758	57.83 ± 17.86***	42.15 ± 21.41^##^	125.0 ± 41.28^∗∗∗, ##,†††^	98.78 ± 49.10***	81.14 ± 37.11***
I	19.82 ± 8.215	41.70 ± 13.06^∗∗∗, ##^	58.39 ± 17.87***	125.3 ± 48.83^∗∗∗, #^	83.46 ± 39.26***	61.13 ± 39.13^∗∗, #^
J	18.98 ± 13.67	30.55 ± 11.18^∗∗∗, ###^	45.82 ± 8.996^∗∗∗, ###^	88.52 ± 19.35***	113.9 ± 53.11***	64.00 ± 35.28***

Low density lipoprotein cholesterol (LDL-C) contents were assayed by assay kits as described in the text.

Values were mean ± SD (*n* = 12) and expressed in mg/dL.

The * indicates a significant difference compared with control group, *P < 0.05, **P < 0.01, ***P < 0.001.

The ^#^ indicates a significant difference compared with model group, ^#^ *P* < 0.05, ^##^ *P* < 0.01, ^###^ *P* < 0.001.

The ^†^ indicates a significant difference compared with PMR group (same dosage level, F compared with C, G compared to D, H compared to E), ^†^ *P* < 0.05, ^††^ *P* < 0.01, ^†††^ *P* < 0.001.

**Table 5:** High density lipoprotein cholesterol (HDL-C) contents in blood samples.

HDL	0D	6D	18D	24D	30D	42D
A	59.94 ± 32.45	94.03 ± 29.81	64.19 ± 24.53	43.87 ± 8.363	56.49 ± 14.07	45.17 ± 10.13
B	87.52 ± 25.16*	231.0 ± 58.91***	82.43 ± 16.51*	103.2 ± 35.14***	120.6 ± 40.79***	159.5 ± 67.11***
C	70.63 ± 25.64	184.7 ± 53.03***	85.64 ± 21.35*	114.6 ± 65.55**	151.1 ± 43.02**	134.6 ± 51.15***
D	68.16 ± 18.51	203.2 ± 54.77***	146.9 ± 29.29^∗∗∗, ###^	91.30 ± 19.25***	151.5 ± 30.35***	154.2 ± 58.52***
E	84.37 ± 20.96*	175.0 ± 52.67^∗∗∗, #^	159.2 ± 36.76^∗∗∗, ###^	104.7 ± 43.40***	164.3 ± 55.30***	162.7 ± 64.15***
F	49.46 ± 13.40^†^	142.6 ± 63.33^∗, ##^	143.8 ± 29.48^∗∗∗, ###,†††^	106.0 ± 47.44***	182.1 ± 79.76***	148.3 ± 50.91***
G	68.74 ± 13.56	157.5 ± 70.00^∗∗, #^	124.7 ± 44.38^∗∗∗, ##^	101.6 ± 34.80***	193.7 ± 70.87^∗∗∗, #^	120.1 ± 54.00***
H	65.84 ± 23.89	240.9 ± 59.83^∗∗∗,†^	131.4 ± 68.25^∗∗, #^	103.5 ± 34.80***	191.2 ± 74.25^∗∗∗, #^	173.9 ± 63.37***
I	57.36 ± 10.28	167.1 ± 49.01^∗∗∗, ##^	65.42 ± 25.85	100.8 ± 41.64***	167.7 ± 68.80***	148.3 ± 86.91***
J	72.06 ± 29.60	148.3 ± 60.49^∗, ##^	113.4 ± 29.27^∗∗∗, ##^	126.1 ± 32.71***	189.2 ± 56.13^∗∗∗, ##^	147.5 ± 76.78***

High density lipoprotein cholesterol (HDL-C) contents were assayed by assay kits as described in the text.

Values were mean ± SD (*n* = 12) and expressed in mg/dL.

The * indicates a significant difference compared with control group, *P < 0.05, **P < 0.01, ***P < 0.001.

The ^#^ indicates a significant difference compared with model group, ^#^ *P* < 0.05, ^##^ *P* < 0.01, ^###^ *P* < 0.001.

The ^†^ indicates a significant difference compared with PMR group (same dosage level, F compared with C, G compared to D, H compared to E), ^†^ *P* < 0.05, ^†††^ *P* < 0.001.

**Table 6:** Very low density lipoprotein (VLDL) contents in blood samples (*μ*mol/L).

Group	0D	18D	42D
A	81.59 ± 12.18	106.9 ± 23.96	125.0 ± 13.12
B	95.15 ± 13.84	92.50 ± 11.50	89.00 ± 13.00**
C	97.48 ± 3.568*	81.80 ± 12.34	79.47 ± 23.60*
D	88.05 ± 13.22	74.28 ± 14.69	97.69 ± 13.09*
F	120.9 ± 4.996^∗∗∗, #,†††^	98.86 ± 14.69	72.58 ± 5.376***
G	121.3 ± 13.33^∗∗,†^	93.03 ± 12.43	96.84 ± 20.52
I	91.12 ± 30.99	76.08 ± 6.439*	104.9 ± 20.62

Very low density lipoprotein (VLDL) contents were assayed by assay kits as described in the text.

Values were mean ± SD (*n* = 12) and expressed in *μ*mol/L.

The * indicates a significant difference compared with control group, *P < 0.05, **P < 0.01, ***P < 0.001.

The ^#^ indicates a significant difference compared with model group, ^#^ *P* < 0.05.

The ^†^ indicates a significant difference compared with PMR group (same dosage level, F compared with C, G compared to D), ^†^ *P* < 0.05, ^†††^ *P* < 0.001.

**Table 7:** Lipid indexes in the liver samples.

Group	TC	TG	HDL-C	LDL-C	AST	ALT
	(mg/dL)	(mg/dL)	(mg/dL)	(mg/dL)	(U/L)	(U/L)
A	66.63 ± 4.093	147.22 ± 6.180	57.79 ± 10.96	10.74 ± 2.186	3286 ± 817.8	1048 ± 344.9
B	100.2 ± 19.22***	200.0 ± 32.56***	76.05 ± 25.82	28.36 ± 12.57**	2539 ± 308.4	1302 ± 519.2
C	105.8 ± 15.01***	197.8 ± 18.56***	79.92 ± 17.93**	28.79 ± 7.821***	2673 ± 428.4	1470 ± 424.5*
D	87.71 ± 17.19**	180.8 ± 15.94***	66.38 ± 17.49	24.49 ± 6.547***	2900 ± 541.4	1394 ± 309.6
E	57.18 ± 6.754^∗∗, ###^	153.6 ± 27.34^#^	84.69 ± 19.50**	24.86 ± 4.385***	1830 ± 288.3^∗∗∗, ##^	1040 ± 490.1
F	66.29 ± 28.08^#,††^	162.1 ± 39.88^†^	88.55 ± 35.98*	38.39 ± 18.53***	1907 ± 582.8^∗∗, #,††^	1268 ± 737.5
G	89.48 ± 18.75**	205.8 ± 29.90***	139.6 ± 24.43^∗∗∗, ##,†††^	48.98 ± 12.02^∗∗∗, #,††^	3661 ± 700.6^##^	1380 ± 693.9
H	69.82 ± 24.30^#^	165.0 ± 32.11	128.8 ± 49.55^∗∗, #^	41.89 ± 16.49^∗∗∗,†^	3579 ± 705.4^##,†††^	1607 ± 592.9*
I	43.67 ± 2.936^∗∗∗, ###^	150.8 ± 18.82^##^	83.79 ± 19.69**	9.990 ± 3.548^##^	3538 ± 304.4^###^	1971 ± 622.0^∗∗, #^
J	44.66 ± 5.379^∗∗∗, ###^	195.8 ± 25.96***	73.20 ± 46.50	15.79 ± 9.505	4244 ± 545.0^∗, ###^	953.5 ± 167.7

All these biochemical indexes were assayed by assay kits as described in the text.

The * indicates a significant difference compared with control group, *P < 0.05, **P < 0.01, ***P < 0.001.

The ^#^ indicates a significant difference compared with model group, ^#^ *P* < 0.05, ^##^ *P* < 0.01, ^###^ *P* < 0.001.

The ^†^ indicates a significant difference compared with PMR group (same dosage level, F compared with C, G compared to D, H compared to E), ^†^ *P* < 0.05, ^††^ *P* < 0.01, ^†††^ *P* < 0.001.

## Abbreviations

AST: Aspartate aminotransaminase
ALT: Alanine aminotransaminase
HDL-C: High density lipoprotein cholesterol
HPLC-DAD: High-performance liquid chromatography coupled with diode array detector
LDL-C: Low density lipoprotein cholesterol
NAFLD: Non-alcoholic fatty liver disease
RPM: Radix Polygoni Multiflori
RPMP: Radix Polygoni Multiflori Praeparata
TC: Total cholesterol
TG: Triglyceride
TSG: 2,3,5,4′-tetrahydroxy-stilbene-2-O-*β*-D-glucoside
VLDL: Very low density lipoprotein
VLDL-C: Very low density lipoprotein cholesterol.
